# “Relapsing–Remitting” Ataxia and Unexpected Brain Imaging in a Child with HIBCH Deficiency

**DOI:** 10.1002/mdc3.14190

**Published:** 2024-08-14

**Authors:** Simone Gana, Gloria Rossetto, Jessica Garau, Valeria Vacchini, Francesca Ferraro, Elisa Rognone, Anna Pichiecchio, Serena Gasperini, Enza Maria Valente, Simona Orcesi

**Affiliations:** ^1^ Neurogenetics Research Center, IRCCS Mondino Foundation Pavia Italy; ^2^ Department of Brain and Behavioural Sciences University of Pavia Pavia Italy; ^3^ Department of Child Neurology and Psychiatry IRCSS Mondino Foundation Pavia Italy; ^4^ Department of Neuroradiology IRCSS Mondino Foundation Pavia Italy; ^5^ Department of Pediatrics Fondazione IRCCS San Gerardo dei Tintori Monza Italy; ^6^ Department of Molecular Medicine University of Pavia Pavia Italy

**Keywords:** HIBCH deficiency, Leigh‐like syndrome, brain MRI, ataxia, whole exome sequencing

3‐Hydroxyisobutyryl‐CoA hydrolase (HIBCH) deficiency, caused by biallelic variants of the nuclear *HIBCH* gene, is a rare disorder of valine catabolism, resulting in the accumulation of toxic metabolites inhibiting mitochondrial function.^1^ To date, HIBCH deficiency has been reported in ~60 cases, most with a Leigh‐like phenotype.^2^ Blood hydroxy‐C4‐carnitine and urinary 2,3‐dihydroxy‐2‐methyl‐butyric and 3‐hydroxyisovaleric acids assist the diagnosis.^2^, ^3^

Here, we report a child with atypical course and unexpected brain imaging of HIBCH deficiency.

The proband is the only daughter born to non‐consanguineous parents, without remarkable personal or familial history.

She never experienced developmental stagnation or regression, seizures or acute encephalopathy. Motor development was delayed (head held at 5 months, sit unsupported at 7, first steps at 25). First words were reported at 24 months.

At 2.4 years, Griffiths Mental Developmental Scale (3rd edition) yielded a general quotient of 51, in line with global developmental delay. Neurological examination showed generalized hypotonia, truncal and gait ataxia and mild upper limb dysmetria (Video 1). Brain MRI disclosed superior cerebellar vermis atrophy (Fig. 1A–E). Exome sequencing identified two novel heterozygous variants in exons 7 and 8 of the *HIBCH* gene (NM_014362.4): c.459 T>A (p.His153Gln) of paternal origin classified as likely pathogenic [ACMG criteria: PM3 (moderate), PM5 (supporting), PP3 (supporting), PM2 (moderate), PP2 (supporting)], and c.663+1G>A of maternal origin, affecting a canonical splice‐site, classified as pathogenic [PVS1 (very strong), PM2 (moderate), PM3 (moderate)].^4^, ^5^ Both variants had never been previously described in patients with HIBCH deficiency nor listed in ClinVar and gnomAD databases.

**Video 1:** At 2.4 years of age, the child was able to remain balanced while sitting unsupported and to stand independently through the squat. However, she stood with feet widely separated and exhibiting truncal swaying. She was able to walk unsupported for short distances but her gait was wide‐based, lurching, and staggering; the trouble was more evident when the patient was asked to turn around and go back.

**Figure 1:**
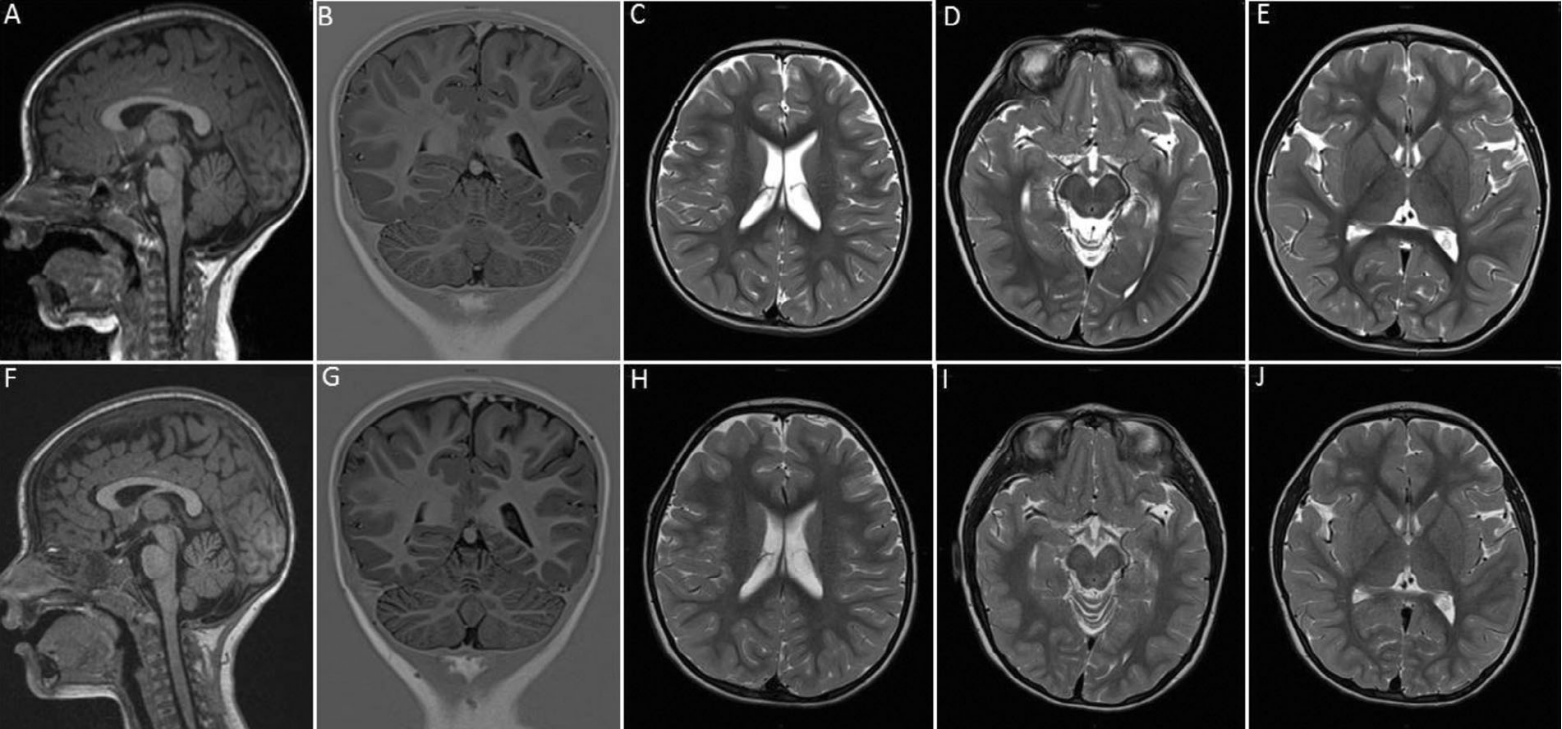
Brain MRI at 2.4 years of age shows mild atrophy of the superior cerebellar vermis (**A**), while the cerebellar hemispheres, white matter, mesencephalon and basal ganglia are normal (**B–E**). Follow‐up brain MRI at 3.4 years reveals progression of the cerebellar atrophy to involve the hemispheres (**F, G**), while the white matter, mesencephalon and basal ganglia are still normal (**H–J**).

Four and 11 months later, she developed two episodes of transient deterioration of motor skills triggered by febrile illnesses (Video 2), but always recovered within 3 weeks, returning to pre‐existing condition.

**Video 2:** At 3.4 years of age, 2 weeks after the febrile illness. Truncal instability worsened, both when sitting and standing, and the patient was unable to walk without assistance.

Brain MRI at 3.4 years revealed mild progression of cerebellar atrophy to both hemispheres (Fig. 1F–J). Metabolic investigations showed a mitochondrial impairment with hyperlactacidemia (3.5 mmol/L) and increased plasmatic levels of alanine, lysine, glycine and proline. Plasma acylcarnitine profile was normal while there was elevated urine excretion of 3‐hydroxyisovaleric acid and trace levels of 2,3‐dihydroxy‐2‐methyl‐butyric acid.

At age 3.7 years, ataxia was stable with mild improvement in motor and language skills (Video 3). A valine‐restricted diet was started: natural protein 1 gr/Kg/die (77 Kcal/Kg, lipids:carbohydrates 34:60%). Diet was combined with oral N‐acetylcysteine (80 mg/kg/die) and Thiamine supplementation (300 mg/die). After 2 months on diet, with good adherence, clinical features remained stable, but metabolic assays revealed normal plasma acylcarnitine and amino acids, no ketonuria or mitochondrial impairment.

**Video 3:** At 3.7 years of age, 3 months after the febrile illness. The patient recovered from the episode by returning to the *status quo ante*.

HIBCH deficiency is characterized by early‐onset progressive developmental delay or by acute regression triggered by febrile infections and metabolic crisis, often leading to early death or severe impairment. Ataxia occurs in ~25% of cases. Early Leigh‐like signal abnormalities of the basal ganglia are hallmarks of the disease, while cortical, white matter, midbrain anomalies and cerebellar atrophy are less frequent.^2^ Though valine‐restricted diet may be effective, there is still no consensus on treatment.^6^

Although the short follow‐up and cerebellar atrophy progression do not allow to exclude the typical neurodegenerative course of HIBCH deficiency, the patient showed a milder clinical expression with global developmental delay and a previously undescribed form of “relapsing–remitting” ataxia, characterized by worsening of motor symptoms after febrile infections followed by recovery, with some neurodevelopmental improvement during remissions.

Genetic testing detected a missense and a splice‐site variant in exons 7 and 8, respectively. Variants within these exons have been reported in patients with heterogeneous presentations.^6^, ^7^ Mild phenotypes of HIBCH deficiency have been described, such as a father and son suffering from mild intellectual disability and non‐progressive ataxia triggered by a febrile seizure during infancy and a few patients presenting with paroxysmal movement disorders, most of them homozygous for the c.913A > G (p.Thr305Ala) missense variant in exon 12.^7^, ^8^, ^9^ Most described variants, especially missense, cluster in this exon with wide phenotypic variability, making genotype–phenotype correlates difficult.^6^

Of note, brain MRI only revealed progressive cerebellar atrophy, while the typical early basal ganglia abnormalities were absent. Only one patient has been described before without this hallmark: a 5‐month‐old boy with severe developmental delay and failure to thrive, featuring widened cerebral sulcus and thinning of the corpus callosum^10^; yet, follow‐up was too short to evaluate progression over time.

As it happens for many rare genetic diseases, these incongruences cannot be explained by specific genotype–phenotype correlates,^2^, ^6^ evoking the occurrence of yet unknown genetic, epigenetic, or environmental modifiers of disease expression.

Although brain MRI scans reveal the distinctive and progressive Leigh‐like abnormalities at onset of symptoms, typically during late infancy or toddlerhood,^2^, ^6^ some reports have shown stabilization or improvement even in imaging findings upon a valine‐restricted diet.^6^, ^7^ Unfortunately, follow‐up of our patient is too short to assess the impact of dietary treatment on clinical and neuroradiological outcomes, although it is worth noting that biochemical parameters improved after 2 months.

Our report highlights that HIBCH deficiency should be also considered in patients without Leigh‐like phenotype, especially in toddlers with developmental delay and non‐progressive ataxia, possibly with a “relapsing–remitting” course. Timely recognition of this potentially treatable disease is critical not only to initiate the dietary intervention as early as possible, but also to allow genetic counseling, prenatal diagnosis and, where appropriate, screening of the wider family.

## Author Roles

(1) Research Project: A. Conception, B. Organization, C. Execution; (2) Statistical Analysis: A. Design, B. Execution, C. Review and Critique; (3) Manuscript Preparation: A. Writing of the First Draft, B. Review and Critique.

S.G.: 1A, 1B, 1C, 3A

G.R.: 1C, 3A

J.G.: 3B

V.V.: 1C, 3B

F.F.: 3B

E.R.: 3B

A.P.: 3B

E.M.V.: 3B

S.O.: 3B

## Disclosures

**Ethical Compliance Statement:** The present work respects ethical guidelines. The author confirms that the approval of an institutional review board was not required for this work. Written consent was obtained for showing the patient's video. We confirm that we have read the Journal's position on issues involved in ethical publication and affirm that this work is consistent with those guidelines.

**Funding Sources and Conflicts of Interest:** The author declares that there are no funding sources or conflicts of interest relevant to this work.

**Financial Disclosures for Previous 12 Months:** The authors declare that there are no additional disclosures to report.
